# Editorial: Plant mineral microbe interactions

**DOI:** 10.3389/fmicb.2025.1677458

**Published:** 2025-09-25

**Authors:** Muhammad Zahid Mumtaz, Tereza Cristina Luque Castellane, Izzah Shahid, Elena Tamburini, Brahim Bouizgarne

**Affiliations:** ^1^Institute of Molecular Biology and Biotechnology, The University of Lahore, Lahore, Pakistan; ^2^College of Agronomy, Gansu Agricultural University, Lanzhou, China; ^3^Plant Biochemistry and Microbiology Laboratory, Department of Agricultural and Environmental Biotechnology, School of Agricultural and Veterinary Sciences, São Paulo State University (UNESP), Jaboticabal/São Paulo, Brazil; ^4^Institute of Multidisciplinary Research in Applied Biology, Public University of Navarra, Pamplona, Spain; ^5^Department of Biomedical Sciences, University of Cagliari, Monserrato, Italy; ^6^Laboratory of Plant Biotechnology (BiotecV), Faculty of Science, Ibn Zohr University, Agadir, Morocco

**Keywords:** soil microbiome, biostimulants and sustainable agriculture, biogeochemical processes, biological nitrogen fixation (BNF), mineral solubilization, plant growth-promoting bacteria

Plant-mineral-microbe interactions are the foundation of soil nutrient dynamics, but their underlying mechanisms and functional integration remain underexplored. Despite growing recognition of the beneficial microbes in the promotion of nutrient uptake through mineral weathering, nitrogen fixation, mineral solubilization, and promotion of plant health, the mechanistic pathways in shaping microbial community composition and functioning are still unclear. The lack of this mechanistic understanding limits our ability to harness these interactions to promote crop productivity under environmental harsh conditions (nutrient deficiency, drought, salinity or degraded soil conditions). Furthermore, conventional agricultural practices often overlook the synergistic relationships between mineral composition and microbial functionality, which limits the bio-based strategies to enhance soil health. Advancing knowledge in plant-mineral-microbe interactions is crucial for developing sustainable, microbe-assisted strategies to improve long-term soil health and agricultural sustainability. Soil health and nutrient cycling are fundamental for sustainable agriculture, directly affecting crop productivity, environmental quality, and ecosystem resilience. Soils, as the largest terrestrial biodiversity reservoir, regulate water quality, carbon sequestration, and greenhouse gas emissions. Therefore, maintaining soil health is essential to address global challenges such as food security, climate change mitigation, and ecosystem conservation. This Research Topic features 15 original research articles alongside a comprehensive review, contributed by over 124 researchers worldwide, demonstrating the expanding global interest in plant-mineral-microbe interactions.

Advancing knowledge in plant-mineral-microbe interactions is crucial for developing sustainable, microbe-assisted strategies to improve long-term soil health and agricultural sustainability. The studies published in this Research Topic cover diverse agroecosystems and management practices, focusing on how organic amendments influence nitrogen metabolism, microbial succession driven by straw retention, and the dynamics of soil organic carbon under different fertilization regimes.

Collectively, these works reveal how soil management shapes microbial communities that drive nutrient cycling and carbon stabilization, processes critical for sustainable crop production. Understanding these underlying mechanisms is essential for developing practices that improve yields while minimizing environmental impacts. Moreover, the research highlights adaptive shifts in microbial functional genes in response to organic inputs, influencing nutrient availability and greenhouse gas fluxes. Advances in soil microbiology and organic matter management demonstrate that integrating these approaches can optimize productivity and sustainability, balancing crop yield goals with ecological stewardship and supporting climate-smart agriculture.

In this context, the review by Pradhan et al. provides a comprehensive overview of the microbial mechanisms driving mineral dissolution, precipitation, and transformation, emphasizing their critical roles in regulating macro- and micronutrient availability in soils. By highlighting microbial activities such as phosphate solubilization, siderophore production, nitrogen fixation, and metal detoxification, this review underscores the vital contributions of microorganisms to soil fertility, sustainable agriculture, and environmental remediation.

Relationships between living microbial biomass, and necromass with soil organic carbon were reported in this topic. Chang et al. contributed critical insights into the effects of long-term organic fertilization on soil carbon dynamics. Over a decade, the application of cattle manure nearly tripled soil carbon stocks compared to conventional chemical fertilization. This increase was primarily attributed to the accumulation of plant-derived compounds and a microbial community shift favoring eutrophic decomposers. The study also reported a marked increase in microbial necromass, confirming the central role of the “microbial carbon pump” in stabilizing soil organic matter. These findings affirm that substrate availability and microbial community structure synergistically promote organic matter persistence, offering practical avenues to enhance soil carbon sequestration in agricultural landscapes. Complementing these findings, Yang et al. examined microbial biomass, necromass, and organic carbon stability in forest soils, highlighting microbial succession as a determinant of soil's capacity to function as a living carbon reservoir. This ecological perspective positions soil as a dynamic and interactive system, where microbial diversity and turnover maintain fertility and enhance carbon sequestration efficiency. The vital contribution of microbial necromass to long-term carbon stabilization informs management practices aiming to increase soil organic matter persistence.

Soil inputs that can drive microbial communities and enhance overall soil health are reflected in multiple interrelated aspects, such as the planting of beneficial species or the application of amendments, compost, and straw materials. Aiming to enhance soil fertility and restructure microbial communities in saline-alkali soils, Song et al., used salt-tolerant plants, and demonstrated their usefulness for improving soil health conditions (salinity, pH, nutrients, enzyme activities, and beneficial microbia diversity). In a comprehensive study by Ma et al., the distinct impacts of various organic amendments, compost, digestate, and straw, on microbial nitrogen cycling were elucidated in anaerobic fluvo-aquic soils. The application of organic matter significantly increased labile carbon fractions, which serve as energy sources for soil microorganisms, and modified the soil functional gene composition related to nitrogen metabolism. Compost-treated plots exhibited the highest abundance of nitrogen-related functional groups, reflecting enhanced microbial activity and nitrogen transformation potential. Although all amendments stimulated denitrification and transient emissions of N_2_O and N_2_ gases while compost significantly reduced N_2_O emissions over the long term. They reported the enrichment of nitrogen mineralizing genes and a concurrent decline in genes involved in dissimilatory nitrate reduction to ammonium and assimilatory nitrate reduction pathways. This metabolic reorganization favors bioavailable nitrogen retention that plants can readily assimilate and promotes nitrogen use efficiency. Intriguingly, biological nitrogen fixation was specifically stimulated in straw-treated soils, underscoring the strategic importance of amendment selection to optimize nitrogen cycling. Key microbial taxa such as *Anaeromyxobacter* and *Lysobacter* emerged as critical players linking microbial community composition to functional soil outcomes to promote environmental sustainability and crop productivity, mitigation of greenhouse gas emissions.

The importance of straw application as a powerfull tool for soil health, acting by changes in microbial communities were exemplfied by four papers (Jia et al., Kimeklis et al., Liu et al., and Ma et al.). Jia et al., investigated the practical agronomic and ecological impacts of straw retention in croplands. Even short-term straw retention significantly increased soil organic carbon and available phosphorus, promoted fungal biomass, and enriched microbial taxa associated with cellulose degradation. Enzymatic activities essential for carbon and nitrogen cycling were elevated, and key genes involved in nitrogen fixation, nitrification, and carbon metabolism were enriched. Together, these changes boosted overall soil functional capacity. However, this practice increased the abundance of phytopathogenic fungi such as *Magnaporthe oryzae*, highlighting the necessity of integrated management approaches that balance agronomic benefits with phytosanitary control. This dual focus exemplifies the complexity of sustainable soil management, where improving soil quality must be harmonized with maintaining plant health.

Using functional metagenomics, Kimeklis et al. identified distinct enzymatic profiles in microbial communities involved in the degradation of straw compared to leaf litter. Their analysis revealed significant functional diversity, particularly enrichment of genes encoding enzymes involved in xylan and pectin degradation, indicating promising biotechnological applications for lignocellulosic residue valorization in agriculture and environmental management. Studies focusing on straw management, such as Liu et al., revealed that incorporation depth and cover crop type distinctly influence bacterial diversity, composition, and community assembly mechanisms. Deep straw incorporation and no-tillage cover enhanced microbial diversity and functional potential, directly contributing to soil resilience and health. Finally, Ma et al., showed that treatment by straw powder, promoted the proliferation of cellulolytic nitrogen-fixing bacteria, including *Clostridium*, and nitrogen-fixing archaea. These results illustrate how targeted agronomic interventions modulate ecological balances and microbial metabolism, supporting sustainable soil function.

Finally, works published in this topic highlighted the importance of bioinoculants which could be represented by (i) beneficial bacteria, either those living in the vicinity of the plant roots (plant growth-promoting bacteria; PGPRs) or those living inside roots (endophites) or and (ii) fungi (especially arbuscular mycorrhizal fungi AMF) in maintaining microbial communities networks (through multiple interactions) and contributing to soil and plant health. Zhu et al. identified promising plant growth-promoting bacteria in the carrot rhizosphere, addressing the limited knowledge on PGPR associated with this crop and highlighting their potential as bioinoculants to stimulate carrot growth. In turn, Li et al. showed that long-term fertilization regimes shape tomato root endophytic communities, with rare microbial taxa playing key roles in network stability and yield improvement. Together, these studies underscore the complementary roles of PGPR in crop-specific rhizospheres and the ecological importance of rare microbial groups in sustaining productivity, offering valuable insights for designing microbial-based strategies to advance sustainable agricultural intensification. Zhang et al. demonstrated that endophytic phosphorus-solubilizing bacteria from tea plants significantly enhanced maize growth and nutrient uptake, particularly phosphorus and selenium. The strain *Pseudomonas fungorum* PMS-05 notably doubled maize dry biomass and increased selenium content, underscoring the synergistic potential of microbial inoculants in optimizing plant nutrition. Such bioinoculants hold promise for tailored nutrient management, especially in soils with specific edaphic constraints. Tang et al. further showed that the efficacy of phosphorus-solubilizing bacterial inoculation depends on fertilization regimes and baseline soil phosphorus levels. Specifically, *Pseudomonas asiatica* JP233 performed optimally in phosphorus-rich soils, modulating the rhizosphere microbiota by enhancing metabolic pathways related to secondary metabolite biosynthesis and phosphorus cycling. Collectively, these findings highlight the need to contextualize bioinoculant application within soil nutrient status and microbial ecology frameworks to maximize agronomic benefits.

Complementing these investigations, the studies conducted by Li et al., Deng et al., and Shi et al. further expand our understanding of microbial biodiversity and its interactions with agricultural management. Li et al. described two new species of the genus *Diachea*, enriching taxonomic and ecological knowledge of slime molds in underexplored regions of China. They identified taxonomic keys and morphological comparisons to support future studies on their distribution and ecological roles. Deng et al. demonstrated how microbial communities associated with the rhizosphere of *Pennisetum alopecuroides* respond distinctly to nitrogen and temperature variations, highlighting the role of soil factors such as pH, potassium, and phosphorus in structuring these communities, revealing regional patterns of microbial cooperation and competition. Shi et al. showed the beneficial effects of organic ground cover on soil enzymatic activity, enrichment of plant growth-promoting rhizobacteria, metabolic shifts linked to sucrose synthesis, and significant increases in fruit yield and biochemical quality. Together, these studies underscore the importance of integrating taxonomy, microbial ecology, and soil management to build more resilient, productive, and sustainability-aligned agroecosystems. Finally, research on arbuscular mycorrhizal fungi (AMF), as illustrated by Yang et al., confirmed their crucial role in regulating heavy metal availability and toxicity in soils, thus protecting plants and mitigating environmental contaminant losses.

Collectively, published work in the current Research Topic reaffirms that sustainable soil management transcends mere input application. It requires embracing the complexity of microbial dynamics, strategically deploying organic amendments and bioinoculants, and integrating agronomic practices that honor the functional complexity of soil ecosystems. A detailed understanding of molecular and ecological mechanisms driving nutrient cycling and soil carbon stabilization opens innovative pathways toward reconciling agricultural productivity with environmental conservation. The agricultural disciplines, especially soil science and agricultural microbiology, are thus at the forefront of guiding the transformation toward more resilient, productive, and sustainable agroecosystems. Continued interdisciplinary research and knowledge transfer will be essential to realize these goals in the face of global challenges posed by climate change, resource limitations, and food security demands. The important insights and advances provided by the studies compiled in this Research Topic have directly contributed to the development and launch of its second volume. This new Research Topic will build upon the foundational knowledge generated here, further advancing our understanding of soil ecosystem processes and management strategies. By addressing emerging challenges and fostering innovative solutions, these ongoing efforts will support the creation of agroecosystems that are both highly productive and ecologically sustainable, capable of withstanding the pressures of a rapidly changing world.

